# Prenatal Neurogenesis in Autism Spectrum Disorders

**DOI:** 10.3389/fchem.2016.00012

**Published:** 2016-03-15

**Authors:** Gaurav Kaushik, Konstantinos S. Zarbalis

**Affiliations:** ^1^Department of Pathology and Laboratory Medicine, University of California at DavisSacramento, CA, USA; ^2^Institute for Pediatric Regenerative Medicine, Shriners Hospitals for ChildrenSacramento, CA, USA

**Keywords:** autism spectrum disorders, neural progenitors, neuronal migration, cerebral cortex, megalencephaly

## Abstract

An ever-increasing body of literature describes compelling evidence that a subset of young children on the autism spectrum show abnormal cerebral growth trajectories. In these cases, normal cerebral size at birth is followed by a period of abnormal growth and starting in late childhood often by regression compared to unaffected controls. Recent work has demonstrated an abnormal increase in the number of neurons of the prefrontal cortex suggesting that cerebral size increase in autism is driven by excess neuronal production. In addition, some affected children display patches of abnormal laminar positioning of cortical projection neurons. As both cortical projection neuron numbers and their correct layering within the developing cortex requires the undisturbed proliferation of neural progenitors, it appears that neural progenitors lie in the center of the autism pathology associated with early brain overgrowth. Consequently, autism spectrum disorders associated with cerebral enlargement should be viewed as birth defects of an early embryonic origin with profound implications for their early diagnosis, preventive strategies, and therapeutic intervention.

## Introduction

Autism spectrum disorders (ASDs, exchangeably used with autism in this review) comprise a complex of neurodevelopmental behavioral anomalies centered on three core deficits: language impairment, social impairment, and limited interests often accompanied by repetitive actions. Typically, ASDs are diagnosed in early childhood exclusively through behavioral evaluation and there is a strong association with gender as boys are affected four times as often as girls (Geschwind, 2011). Approaches to treatment are focused on improving social skills as well as language and communication, and there are no medications currently available to alleviate the core symptoms. Recent decades have seen a continuous rise in prevalence of ASDs in several countries (Palmer et al., 2010; Parner et al., 2011; Paula et al., 2011). This trend appears not to be uniform though, as a recent study in Sweden reported no changes in autism prevalence over a 10 year period (Lundström et al., 2015) and part of the rise has been attributed to the expansion of diagnostic criteria and increased reporting. In the United States in particular, where this worrisome trend has been closely monitored (Kong S. W. et al., 2012; Blumberg et al., 2013), the CDC reports (Blumberg et al., 2013) the number of diagnosed cases has increased sevenfold over the last 20 years. Currently, the prevalence in the US is close to one in 50 (Blumberg et al., 2013).

Formidable advances in untangling the complex genetic etiology of ASDs have been recently enabled by the assembly of large DNA collections of affected individuals and their families, particularly the Simplex Collection (SSC; Fischbach and Lord, 2010), and progress in DNA sequencing technologies (O'Roak et al., 2011, 2012; Iossifov et al., 2012, 2014; Neale et al., 2012; Sanders et al., 2012). These studies have identified two intriguing aspects of autism genetics. First, causative genes in ASDs are characterized by an enormous diversity involving hundreds and possibly up to a thousand genes. Second, the genetic contribution largely stems from rare variants in protein-coding genes, none of which accounting for more than 1% of the total number of cases. This complex landscape of autism genetics apparently underlies also the great heterogeneity of endophenotypes, one of the major challenges in identifying and defining neuropathological abnormalities that are shared across the autism spectrum (Happé et al., 2006). However, as the understanding of genetic causes increases and data on the spatiotemporal expression and interaction of causative autism genes, and the proteins they encode, become available the opportunity arises to subclassify autism cases and recognize the respective underlying pathology. For instance, several known autism factors, such as Nrxn, Nlgn, and Shank have a defined role in synaptic cohesion and function (Toro et al., 2010). Coexpresssion network analyses of the encoding genes, implicate early postnatal stages associated with neuronal differentiation and synaptic maturation as probable points of vulnerability to the development of ASDs (Parikshak et al., 2013). In contrast, developmental regulators found to be causative in autism, such as CHD8, TBR1, and FMR1, apparently form distinct networks that converge on prenatal coexpression modules of developing cortical projection neurons (Parikshak et al., 2013; Willsey et al., 2013).

## A subset of ASD cases displays alterations in overall and regional brain growth trajectories

The search for genetic determinants in ASDs has been accompanied by an equally intense search for neuropathological changes in the autistic brain. However, the survey for consistent neuroanatomical changes across the autism spectrum has been hampered by the heterogeneity of cases, rooted in very different causes, and often associated with diverse comorbidities. Nonetheless, in particular magnetic resonance imaging (MRI) studies and postmortem analysis of brains of affected children have produced convincing evidence that cerebral overgrowth in early childhood is a defining feature in a subset of autism cases (Courchesne et al., 2001, 2003; Sparks et al., 2002; Hazlett et al., 2005). The size of this subset varies among studies, as small proband cohorts appear to introduce considerable variability, but may comprise approximately 20% of all ASD cases (Fombonne et al., 1999; Nordahl et al., 2011). In these cases, normal or even slightly reduced cerebral size at birth is followed by a period a rapid growth within the first year, followed by a period of slowed growth after 2–3 years of age (Redcay and Courchesne, 2005; Courchesne et al., 2007, 2011a). The cerebral cortex is the primary source of overgrowth; however, other forebrain structures exhibit enlargement as well, most prominently the amygdala, an important center of emotional learning and memory processing (Sparks et al., 2002; Schumann et al., 2004). Intriguingly, several studies reported that cerebral overgrowth is not uniform, but rather regional with frontal and temporal cortices predominantly affected while other aspects are not affected (Carper et al., 2002; Kates et al., 2004; Hazlett et al., 2005; Bloss and Courchesne, 2007; Schumann et al., 2010). Interestingly, the higher-order cognitive processes affected in ASDs such as language, sociability, and emotion correlate well with these areas of pronounced hyperplasia suggesting that pathological regional cortical expansion and autism core deficiencies are tightly linked (Baron-Cohen and Belmonte, 2005; Amaral et al., 2008). Furthermore, brain overgrowth appears to indicate particular severity of symptoms and is associated with regression, the increased loss of social an/or communicative abilities later in life (Nordahl et al., 2011).

The inheritance patterns of megalencephaly remain uncertain and their association to ASDs are complicated by the presence of megalencephalic children without ASD symptoms. A recent study demonstrated partial inheritance of brain size in families with members on the spectrum, suggesting that enlarged brain size predisposes affected individuals and likely presents an endophenotype of ASD (Froehlich et al., 2013). Another study found that ASD cases with macrocephaly were more likely to have 1st degree relatives with benign/non-ASD-related macrocephaly (Fidler et al., 2000). ASD cases with brain sizes over the 75th percentile (enlarged, but not classically defined as macrocephaly, which is >97th percentile) had greater impairments in adaptive functioning and had higher rates of immune dysfunction, as well as their 1st degree relatives (Sacco et al., 2007). In these cases, macrocephaly was generally found to associate with generalized somatic overgrowth, a finding replicated in a separate study in boys only (Campbell et al., 2014).

Intriguingly, developmental brain enlargement appears to be a point of convergence for environmental ASD risk factors as well, particularly cases associated with maternal inflammatory response, referred to as maternal immune activation (MIA). Studies have shown a positive association between early brain overgrowth and maternal inflammation in a subset of ASD cases, suggesting a link between inflammatory states and prenatal brain development in ASD (Sacco et al., 2007; Nordahl et al., 2013). Research into MIA associated with autoantibodies directed against fetal brain proteins has provided additional insight in this context, as targeted fetal brain proteins include factors critical for neural progenitor proliferation and differentiation (Braunschweig et al., 2013). Moreover, intraventricular injections of maternal autoantibodies into mouse embryos result in enhanced neural progenitor proliferation during development, as well as increased neuronal cell size and larger brains in adults (Martínez-Cerdeno et al., 2014).

## Altered neurogenesis in ASDs

While early brain overgrowth has been recognized as a key feature in a subset of young children on the autism spectrum, the causes of this enlargement have not been exhaustively explained even though an excess in excitatory projection neurons appears reasonable. Projection neurons comprise the vast majority of neurons in the cortex (80%) and could with their dendrites, synapses, axons, and myelin, produce the increase in gray and, occasionally, white matter volumes reported in young autistic children. Indeed, this assumption has been recently considerable strengthened through work assessing and comparing neuron numbers in autistic and normal children postmortem, which found 67% more neurons in the prefrontal cortex of autism cases (Courchesne et al., 2011b). Drastically altered neuron numbers in postnatal life can be indicative of changes in developmental neurogenesis, which in humans occurs largely during late embryonic and early fetal development, from gestational weeks 7–20. As cell generation dominates cell elimination by at least a factor of 100 in the developing brain (Rakic and Zecevic, 2000), the contribution of cell death to megalencephaly during this period appears negligible.

Proper developmental neurogenesis depends upon the tightly regulated balance between symmetric divisions of radial glial cells, that produce additional radial glial cells, and asymmetric divisions, that generate intermediate progenitors and postmitotic cells. Newly formed projection neurons, predominantly generated by the symmetric divisions of intermediate progenitors in the subventricular zone, migrate radially outward to establish the six-layered neocortex by successively forming first the inner and then the outer laminae (Gupta et al., 2002; Nadarajah and Parnavelas, 2002; Kriegstein and Noctor, 2004). As symmetric mitoses drive lateral expansion and asymmetric mitoses produce radial growth of the cortical sheet, natural or pathological shifts in the mode of neural progenitor division have the capacity to direct cortical morphological outcomes and brain growth in general (Fish et al., 2008). Consequently, changes in developmental neurogenesis could, in addition to altered numeric output, generate structural changes reflecting disturbances in neuron generation. Indeed, such evidence has been produced by several studies. A first inquiry in this area identified in four out of six ASD cases focal cortical anomalies of misaligned projection neurons and marginal zone heterotopia (Bailey et al., 1998). A 2007 study using both MRI and postmortem histological analysis identified cortical lamination abnormalities and supernumerary neuronal foci in seven out of eight examined ASD cases (Hutsler et al., 2007). Wegiel et al. report in 12 out of 13 of the autistic brains examined, findings of multifocal cerebral dysplasia reflective of broad dysregulations of neurogenesis, neuronal migration and maturation in addition to subcortical, periventricular, hippocampal, and cerebellar heterotopias (Wegiel et al., 2010). The analysis of seven additional affected individuals revealed foci of cortical thinning, classified as focal cortical dysplasia, affecting particularly the frontal lobes (Casanova et al., 2013). These findings were further corroborated by a more recent study that used molecular markers to identify widespread patches of disorganized cortical layering in 10 out of 11 examined cases (Stoner et al., 2014). Focal cortical dysplasia, a well-recognized cause of intractable epilepsy, provide a possible explanation for the high prevalence of epilepsy as a comorbidity in autism, with co-diagnoses reaching up to 40% of autism cases (Danielsson et al., 2005; Mouridsen et al., 2011). Collectively, these studies provide strong support for the concept of altered neural progenitor proliferations as a key event in pathological outcomes characteristic of a subtype of autism cases, associated with developmental megalencephaly and focal cortical lamination defects. The cellular substrate of megalencephaly is likely provided by cortical projection neurons, but apparently not by a concomitant increase in glia cells (Courchesne et al., 2011b; Morgan et al., 2014) even though astrocytes are generated by the same progenitor pool that generates projection neurons. Similarly, cerebral white matter, largely composed of myelinated axons of cortical projection neurons and glia, has not been found to be significantly increased in young children on the spectrum with megalencephaly (Hazlett et al., 2005; Friedman et al., 2006).

## Neural progenitors and ASDs

**Figure 1:**
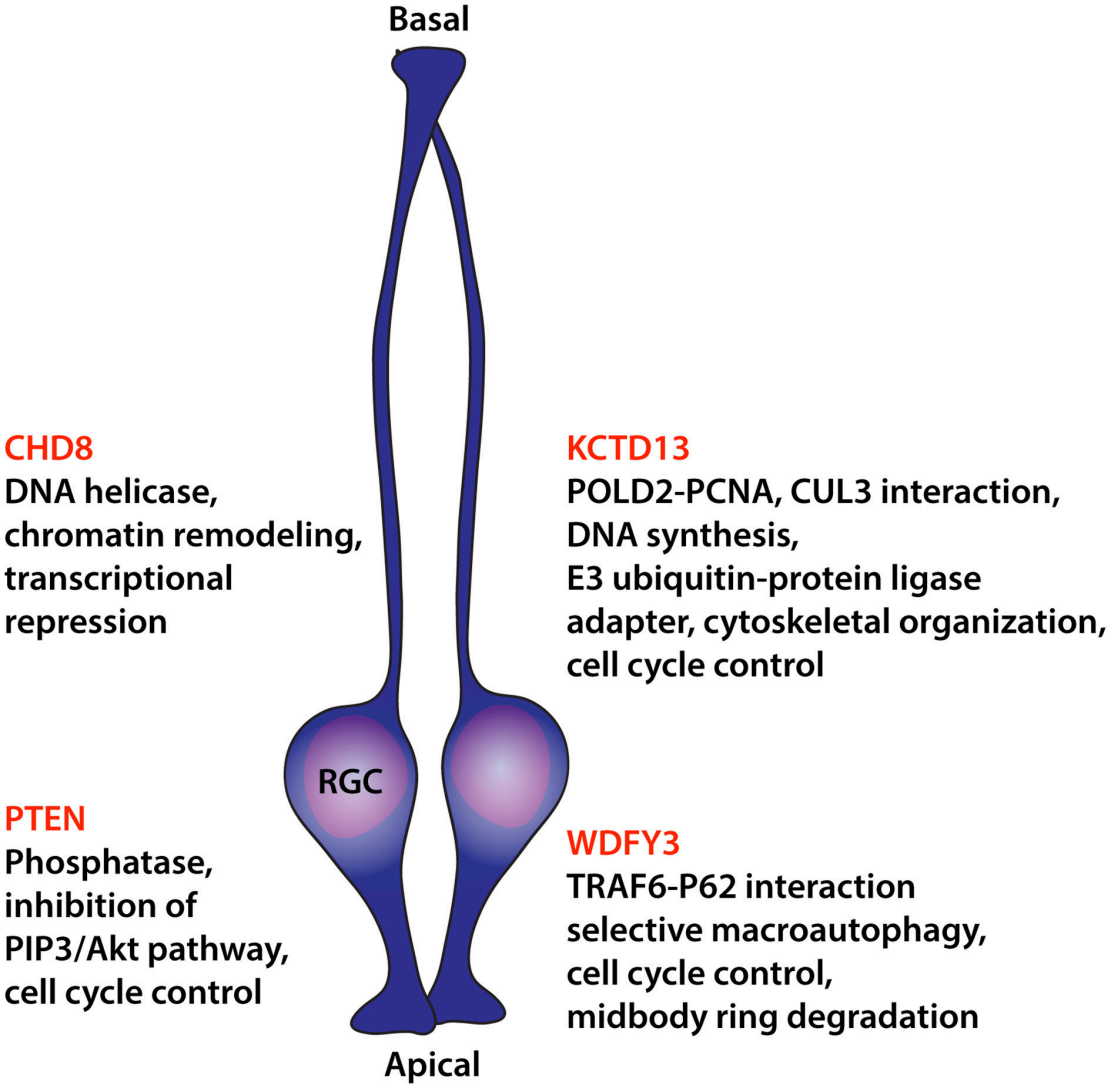
**Diagram summarizing findings on causative ASD molecules that act on neural progenitor proliferation**. Listings include recognized molecular functions potentially underlying control over cellular division. RGC, radial glial cell.

In recent years, insight into the developmental neurogenic basis of brain enlargement in autism has been substantially aided by the discovery of genes causative in autism and encoding regulators of neural progenitor proliferation (Figure 1). Phosphatase and tensin homolog (*PTEN*) gene was the first gene unambiguously linked to macrocephaly in autism (Goffin et al., 2001; Butler et al., 2005; Buxbaum et al., 2007; Varga et al., 2009). The gene was initially identified as a tumor suppressor, frequently mutated in a variety of cancers and apparently regulating cellular proliferation and cell cycle arrest through the phosphatidylinositol 3-kinase (PI3K)/Protein kinase B (PKB/Akt) pathway (Zhao et al., 2004). Nestin-cre mediated conditional inactivation of *Pten* in mice closely replicated the megalencephaly observed in humans, a phenotype largely driven by an increase in neural progenitor proliferation even though a reduction in apoptosis was observed as well (Groszer et al., 2001).

Naturally, the detailed study of altered neural progenitor proliferation and developmental neurogenesis in humans is made impossible by ethical and practical considerations. All insight depends on retrospective examination, ideally of young brains, through MRI and the few tragic cases that become accessible through tissue donation and postmortem analysis. However, animal models that replicate aspects of human phenotypic alterations and are experimentally accessible through a wide variety of tools can provide recourse to a deeper understanding of pathological neurogenesis in autism. Genetic zebrafish and mouse models have dominated recent advances in this area. For instance, a 2012 study in zebrafish to ascertain the neurodevelopmental effects of the 29 genes involved in 16p11.2 copy number variants provided interesting results with respect to autism-relevant changes in brain size (Golzio et al., 2012). 16p11.2 copy number variation is causative in neurocognitive disorders with curiously both duplications and deletions resulting in ASDs (Weiss et al., 2008; McCarthy et al., 2009). The neuropathological outcomes are opposite though, with deletions leading to macrocephaly and duplications to microcephaly in humans. These phenotypic outcomes were closely replicated in the respective zebrafish experiments with either effect solely attributed to potassium channel tetramerization domain containing-13 (*KCTD13*) while shRNA-mediated knockdown of *Kctd13* in mouse embryos increases neural progenitor proliferation. The same study also reported on an ASD proband with a 9 kbp deletion spanning *KCTD13* exons 3–5 further confirming the critical involvement of this gene in autism. KCTD13 is a nuclear protein directly interacting with DNA polymerase delta subunit 2 (POLD2) and proliferating cell nuclear antigen (PCNA) and consequently involved in DNA synthesis and replication (He et al., 2001). While the mechanism by which KCTD13 influences progenitor proliferation remains uncertain its interactions with POLD2 and PCNA suggest control over cell cycle progression. This assumption is further strengthened by the functional interaction of KCTD13 with the scaffold protein cullin 3 (CUL3) (Lin et al., 2015). CUL3 is a component of E3 ubiquitin-protein ligase complexes that among other roles ubiquitinate and degrade cyclin E, thus directly controlling cell cycle progression (Singer et al., 1999). Intriguingly, also for *CUL3* two nonsense *de novo* alleles in ASD cases were identified (Kong A. et al., 2012; O'Roak et al., 2012) that specifically disrupt CUL3 interaction with KCTD13 (Lin et al., 2015).

*CHD8*, which encodes a chromatin regulating DNA helicase, has emerged as a key gene in ASDs with 9 *de novo* likely gene-disrupting mutations identified in the SSC (O'Roak et al., 2012; Iossifov et al., 2014). A broader search for disruptive alleles that included in addition to ASDs also children exhibiting developmental delay identified 12 additional *CHD8* truncating mutations (nonsense, frameshift, and canonical splice site) (Bernier et al., 2014). Intriguingly, 80% of affected individuals carrying likely causative *CHD8* alleles exhibit macrocephaly, a significantly higher percentage than overall macrocephaly presentation in ASD cases of the SSC without *CHD8* mutations, possibly defining a subtype. For two of the affected children, head circumference growth trajectories were available and confirmed in the first 2 months unusual growth of the orbital frontal head. In these cases, increased head circumference persisted throughout early childhood at or above the 97th percentile. Modeling of the *CHD8* phenotype in zebrafish embryos by gene knockdown, produced megalencephalic morphants exhibiting an increase in the *otx2*^+^ forebrain/midbrain neural progenitor population and pointing at overproliferation of neural progenitors as the main driver of the observed brain size increase (Bernier et al., 2014; Sugathan et al., 2014).

*WDFY3*, similarly identified through surveys of causative *de novo* variants in ASD from the SSC (two cases; Iossifov et al., 2012, 2014), is an additional gene implicated in megalencephaly and altered neural progenitor proliferation. Previous work has characterized WDFY3 as a scaffold protein, required for the selective autophagic degradation of macromolecular components such as aggregation-prone proteins in a process described as selective macroautophagy (Filimonenko et al., 2010). Loss of *Wdfy3* in mice has profound effects on neural progenitor proliferation and neuronal migration (Orosco et al., 2014). *Wdfy3* mutant mice exhibit larger brains as a consequence of a relative change in the mode of radial glia mitoses from asymmetric to symmetric. This shift in the proliferative mode of radial glial cells increases the progenitor population and, as a consequence, cerebral size. Furthermore, since progenitor expansion and cortical neurogenesis proceeds in a spatiotemporal gradient during development, initiated anterolaterally and concluded posteromedially (Caviness et al., 2009), the proliferative changes in *Wdfy3* mutant mice disproportionally affect the anterolateral areas of prolonged neurogenic period. This important aspect replicates similar findings reported in some MRI studies of brains of affected children, in which frontal and temporal cortices were predominantly affected by size increase (Carper et al., 2002; Kates et al., 2004; Hazlett et al., 2005; Bloss and Courchesne, 2007; Schumann et al., 2010). Importantly, this regionality in cerebral overgrowth may be intimately linked to the core behavioral pathology in affected children. In humans these areas contain the orbitofrontal and ventrolateral prefrontal cortex, the superior temporal sulcus, and the insula of the temporal cortex, key regions in determining social value, reward, reinforcement, interoception, and emotional processing that are centrally affected in ASDs (Pelphrey and Carter, 2008; Redcay, 2008; McPartland et al., 2011; Gotts et al., 2012; Gasquoine, 2014).

The mechanism by which Wdfy3 exerts control over cellular division remains uncertain, but expression analysis revealed its specific upregulation in neural progenitors during mitosis (Orosco et al., 2014), possibly acting in the autophagic degradation of specific proteins instrumental in cell cycle control, as the cell cycle in *Wdfy3* mutant mice is shortened. Interestingly, WDFY3, the autophagy receptor SQSTM1/p62, and the ubiquitin E3 ligase TRAF6 form a complex that ensures the ubiquitination and efficient clearance of midbody ring derivatives after mitosis (Isakson et al., 2013). Apparently, retention of the midbody ring in daughter cells is associated with the maintenance of undifferentiated characteristics in stem and cancer cells (Ettinger et al., 2011; Kuo et al., 2011), providing a possible explanation for the increase in the progenitor population of *Wdfy3* mutants.

## Conclusion and future steps

Evidence accumulated over the last few years strongly supports the notion that a subgroup of children on the autism spectrum may exhibit unusual brain enlargement soon after birth. This megalencephaly affects predominantly the cerebrum and is associated with an increase in the number of neurons in affected areas. The most plausible explanation for this unusual cerebral overgrowth appears to be an increase in the proliferation of neural progenitors during embryonic development, which has been convincingly corroborated by animal models. Intriguingly, developmental megalencephaly may be a point of confluence for both genetic and environmental factors in ASDs as work on maternal inflammation during pregnancy suggests. Thus, early brain overgrowth in autism has to be viewed as a birth defect rooted in the altered proliferative program of neural stem cells during the earliest stages of developmental neurogenesis. Importantly, as environmental factors appear to be involved in autism development as well, nutrients, drugs, pollutants, toxins, pathogens, and other maternal exposures should be carefully evaluated for their possible contributions to altered neurogenesis in the embryo/fetus and associated megalencephaly in autism. Harmful exposures, assessed and recognized by *in vitro* assays or suitable animal models, could be individually targeted through preventive strategies with positive epidemiological effects. The prospect that neural progenitors are centrally involved in pathological changes that define a subset of autism cases opens up the possibility for the development of novel biomarker-based diagnostics exploiting proliferative changes of stem cell compartments. Diagnostic markers, validated in peripheral samples, such as blood, could be applied early in life and possibly supplement the current regime of behavioral evaluation for added diagnostic certainty. At risk children, recognized through biomarkers, could be submitted earlier to behavioral intervention before treatment-resistant brain defects develop. In addition, ASDs rooted in altered developmental neurogenesis may define a specific subtype within the spectrum with special considerations with respect to comorbidities and trajectories, requiring distinct pathways to intervention.

## Author contributions

All authors listed, have made substantial, direct and intellectual contribution to the work, and approved it for publication.

### Conflict of interest statement

The authors declare that the research was conducted in the absence of any commercial or financial relationships that could be construed as a potential conflict of interest.
